# Case Report: Desmoid fibromatosis in the mediastinum of a 6-month-old toddler, what to do?

**DOI:** 10.3389/fsurg.2022.1007760

**Published:** 2023-01-30

**Authors:** Santiago A. Endara, Gerardo A. Dávalos, Gabriel A. Molina, Christian A. Armijos, D. Sebastian Narvaez, Nelson Montalvo

**Affiliations:** ^1^ Department of General Surgery, Division of Cardiothoracic Surgery, Hospital Metropolitano, Quito-Ecuador; ^2^ Universidad San Francisco de Quito, Quito-Ecuador; ^3^ Department of Internal Medicine, Imaging and Interventional Radiology Service, Hospital Metropolitano, Quito-Ecuador; ^4^ Universidad Internacional del Ecuador-Hospital Metropolitano PGY1 General Surgery, Quito-Ecuador; ^5^ Department of Internal Medicine, Pathology Service, Hospital Metropolitano, Quito-Ecuador

**Keywords:** desmoid fibromatosis, mediastinal mass, desmoid tumor, desmoid disease, pediatric

## Abstract

Desmoid fibromatosis is a rare, aggressive borderline lesion arising from soft tissues. Treatment will depend on the structures that the tumor has involved. Surgery with negative margins is the recommended strategy as it can usually achieve disease control; however, the tumor's location sometimes does not allow it. Therefore, a combination of medical therapies along with strict surveillance is crucial. We present the case of a 6-month-old boy with a chest mass. After further evaluation, a rapidly growing mediastinal mass involving the sternum and costal cartilage was detected. Desmoid fibromatosis was the final diagnosis.

## Introduction

Desmoid fibromatosis is a rare soft tissue neoplasm that does not metastasize but presents an aggressive growth and local invasion (1). It can affect all age groups and occur throughout the body (1, 2). In children, there is limited information on managing these complex tumors. We present the case of a young boy in whom a rapidly growing mass was detected in his chest. Desmoid fibromatosis was the final diagnosis.

### Case presentation

Patient is a 6-month-old who was born with clinodactyly in both hands, and without a history of familial adenomatous polyposis. He presented with a two-month history of a small, hard, nonmobile, and painless mass in his chest. The mass was initially small and did not disturb the child's development. However, a couple of months later, as the child began to crawl, his mother noticed that he had some discomfort and realized that the mass had doubled its size, so she visited his pediatrician.

**Figure 1:**
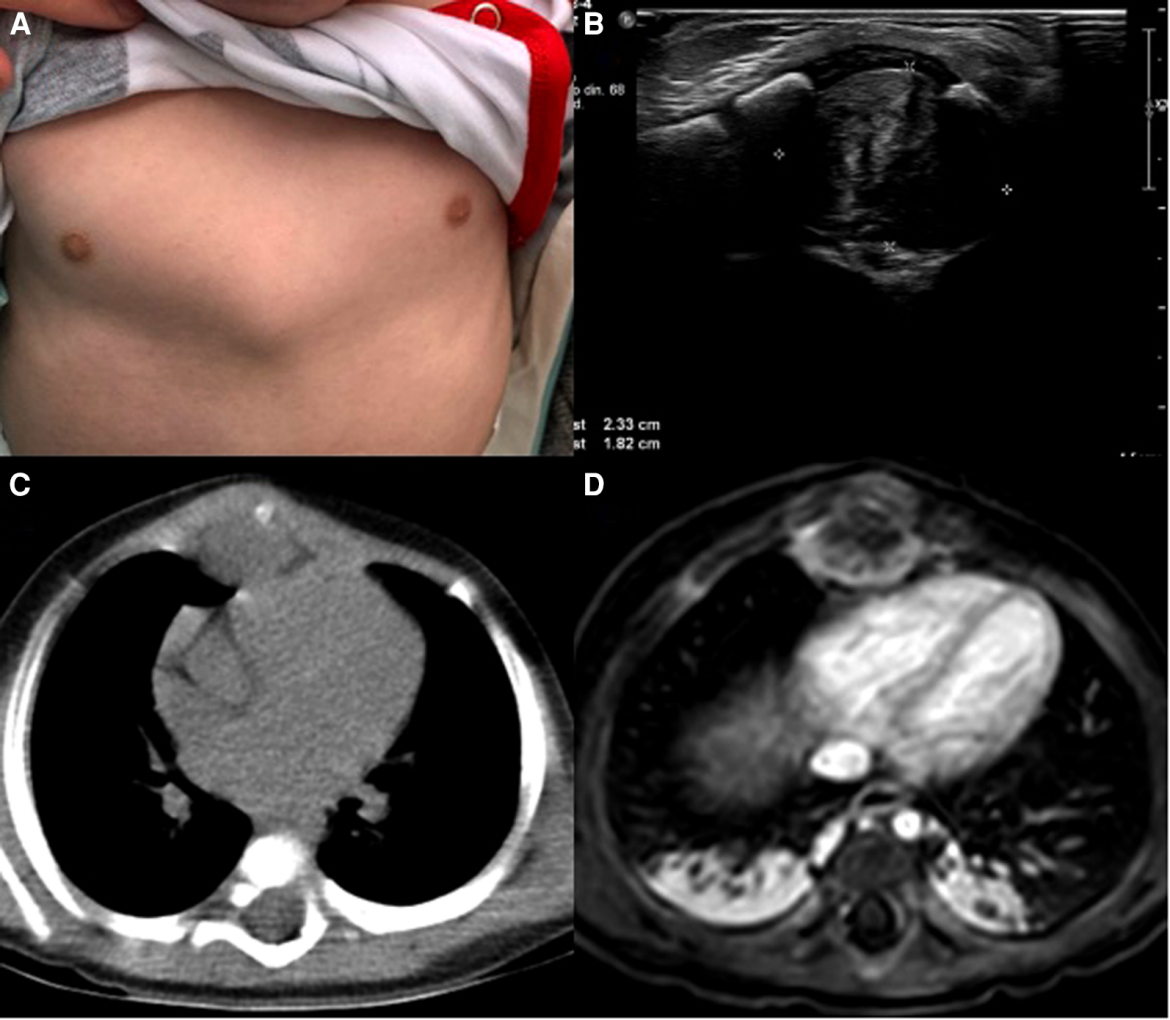
**(A**) chest mass, **(B**) ultrasound, echogenic mass in the anterior mediastinum. (**C**) CT, The mass is in close contact with the xiphoid process. (**D**) MRI, the mass is near the pericardium.

On clinical examination, an otherwise healthy young boy was found. Over the xiphoid process, a 2 × 2 cm solid mass with rounded edges was discovered. The mass was attached to the xiphoid process and nearby structures. With these findings, an ultrasound was requested, discovering an echogenic mass measuring 2.3 × 1.8 cm without vascularization and cardiac involvement (Figures 1A,B).

Due to this, a chest computed tomography (CT) revealed the presence of a 3.5 × 2 cm soft tissue density mass in the anterior mediastinum. It infiltrated the subcutaneous tissue and involved the ribs and sternum. Magnetic resonance imaging also proved that the pericardium was not compromised (Figures 1C,D).

**Figure 2:**
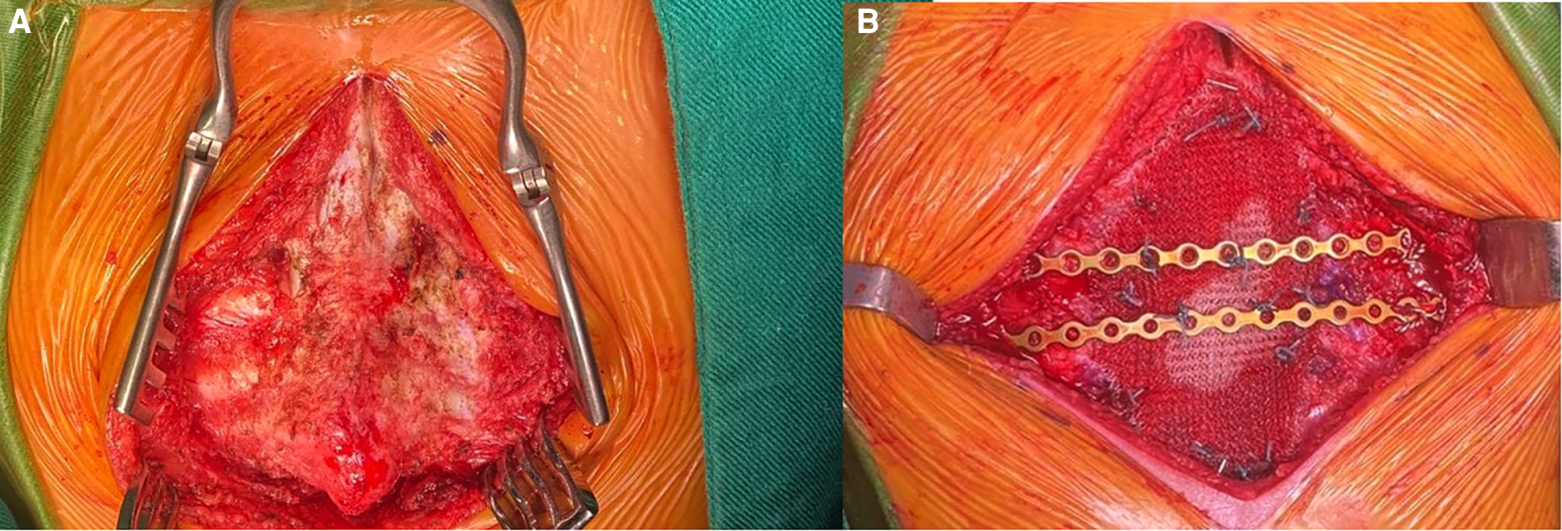
(**A**) Mass invading the costal cartilages and sternum. (**B**) Reconstruction with prosthetic material.

With these findings, surgery was decided, and after partial distal sternotomy, the 3.5 × 2 cm solid whitish mass was discovered; it was in contact with the diaphragm, xiphoid process, and costal cartilages. Biopsy was taken for intraoperative frozen section; however, the results were inconclusive, malignancy was not confirmed, but atypia could be observed; because of this, and considering the risk of malignancy, complete resection of the mass, including the distal third of the body of the sternum, xiphoid process, diaphragm, and four bilateral costal cartilages was completed. A bioabsorbable mesh was used to repair the diaphragmatic defect and rigid fixation was placed on the ribs with stainless steel wires to reconstruct the thoracic wall, since bioabsorbable prosthetic material was unavailable; drains were placed, and the procedure was completed without complications (Figures 2A,B).

**Figure 3:**
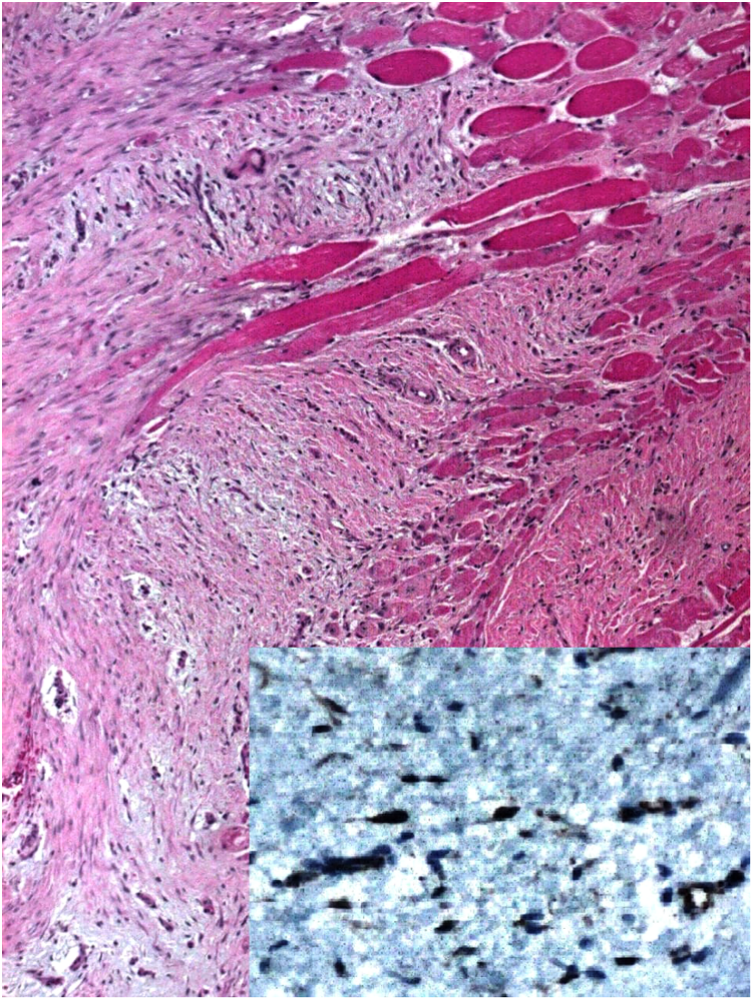
Pathology HE 4X, fusiform cells without nuclear hyperchromatism, hypocellular, and hyalinized areas are seen. Immunohistochemistry positive for Beta-catenin (Inset 40x).

Pathology reported a proliferation of fusiform cells without nuclear hyperchromatism or mitosis; some areas showed multiple nodular, hypocellular, and hyalinized areas surrounding and invading muscle fibers. In addition, surgical margins were compromised on the diaphragmatic side. Immunohistochemistry was positive for Beta-catenin in the nuclear cells and negative for CD34, Desmin, and S100. Desmoid fibromatosis (DF) was the final diagnosis (Figure 3).

His postoperative course was uneventful. He was discharged on postoperative day five, and he is on close follow-ups a year after surgery, there has been no sign of recurrence or complications, and this disease has not impaired his development. He is entirely normal at this time.

## Discussion

DF is an extremely infrequent tumor (2 to 4 cases per million per year) (1, 2). It's categorized as a slowly growing borderline tumor with low malignant potential formed by the monoclonal proliferation of fibroblasts in muscles, tendons, and ligaments (2, 3). This proliferation can lead to local aggressiveness that, depending on its location, can severely affect or impair vital organs, leading to functional impairment and life-threatening conditions (1, 3). Although the pathological pathways are still under investigation, DF is believed to appear from the mesenchymal stromal cells with mutation of the CTNNB1 gene in adults (the *β*-catenin gene) (1, 4). In children, it's associated with AKT (31%), BRAF (19%), and p53 (9%) mutations, in addition to the CTNNB1 (3–5).

DF can present in two ways; the sporadic form, which more frequently affects women, and the condition associated with familial adenomatous polyposis (FAP) (1, 6). DF clinically appears as a firm, often asymptomatic, slow-growing mass (2, 3). Its course can be indolent (5 to 10% of the cases) or invade nearby structures (3, 4). In children, DF primarily affects the head and neck (1). In adults, the trunk and limbs are more affected (1, 2). In our case, the patient presented with a mass that affected his thoracic wall. Treatment will depend on the course of the tumor; a conservative approach or “watch-and-wait” strategy has been suggested if the tumor doesn't show progression (6, 7). However, surgery is recommended if the tumor is over 5 cm, located in the head or neck, and affects younger patients (<10 years) (3, 5). As we did in our case.

Treatment will be based on the symptoms and the tumor growth, especially in critical locations where an earlier surgical decision may potentially prevent a higher risk of morbidity (9). The primary goal should be a wide (R0) microscopic margin resection; however, in problematic locations like the skull base or when functions or cosmetics is an issue, positive (R1), microscopic margins can also be accepted (8–10). Treatment options other than surgery may be preferred when positive microscopic margins are found (10). If an R1 resection is achieved after the initial surgery, in that case, neither perioperative radiotherapy nor reoperation is necessarily required since the risk of a local recurrence when we compare surgery alone and perioperative radiotherapy is not statistically significantly different (11). In contrast, the morbidity of the two combined modalities is higher (10, 11).

Even with successful surgery the risk of relapse is high especially in children where pediatric DF is associated with a high recurrence rate (24%–76%) (5, 6). Therefore, other treatments have also been proposed (8, 9). Another thing to consider is reconstruction in pediatric patients, as children have unique characteristics related to growth; therefore, the prosthetic material for reconstruction should be chosen carefully. Bioabsorbable materials should be used to avoid complications (8, 9). Since these materials were not available to us, we had to use rigid fixation.

Other therapies include radiation therapy as an adjuvant treatment; however, the risk of adverse effects, such as fibrosis, paresthesia, skin ulcers, and, in children, a significant risk of long-term functional sequelae and radiation-induced cancers, must be taken into account. Several cytotoxic and non-cytotoxic drug treatments have been proposed for desmoid tumors, and several have had partial or complete responses in multiple trials (11, 12). Newer treatments, such as tyrosine kinase inhibitors of vascular endothelial growth factor receptors, have also arisen as potential therapies in the future (6, 7). However, each case must be individualized. In our case, surgery was completed as the mass had aggressive behavior, but we could not achieve negative margins; therefore, even though radiotherapy, chemotherapy, anti-hormonal therapies, and tyrosine kinase inhibitors were considered. After exposing all the treatments with their benefits and risk to the child's parents, and multiple discussions with a pediatric oncologist, surveillance was decided.

When facing a desmoid tumor and its treatment, especially in pediatric patients, there are still many gray areas between surgery, observation, and medical therapies. Deciding between them is not always easy, as we should always avoid or limit any possible harm from the disease and our treatments. Still, quality of life must be the single central dimension to weigh when facing these tumors, a decision that must always be made following the wishes of the parents and the child. This article has been prepared according to CARE Guidelines (13).

## Patient perspective

His parents found it difficult at first to accept the disease, especially after the pathology and the positive margins of the tumor were explained to them. However, after the patient's successful recovery and normal development, they have come to accept its nature. They have changed their approach and have learned to accept the nature of the disease in a way that has surprised the entire medical team. We are grateful to the parents for allowing us to treat the patient, and we are on close watch.

## Conclusion

The management of DF is complex and even with treatment, it can still behave like an aggressive disease. Further research is needed to determine which patients will benefit from surgery, medical therapies, or close surveillance. We will always have to weigh the risk of treating the disease with all the complications and recurrences that it entails or waiting and losing the opportunity to offer a chance of a complete cure.

## Data Availability

The original contributions presented in the study are included in the article/Supplementary Material, further inquiries can be directed to the corresponding author.

## References

[1] Miyashita H Asoda S Soma T Munakata K Yazawa M Nakagawa T Desmoid-type fibromatosis of the head and neck in children: a case report and review of the literature. J Med Case Rep. (2016) 10:173. 10.1186/s13256-016-0949-9 27286970 PMC4902910

[2] Fortunati D Kaplan J López Martí J Ponzone A Innocenti S Fiscina S Desmoid-type fibromatosis in children. Clinical features, treatment response, and long-term follow-up. Fibromatosis tipo desmoide en niños. Características, respuesta al tratamiento y seguimiento a largo plazo. Medicina (B Aires). (2020) 80(5):495–504. PMID: 33048794 33048794

[3] Paul A Blouin MJ Minard-Colin V Galmiche L Coulomb A Corradini N Desmoid-type fibromatosis of the head and neck in children: a changing situation. Int J Pediatr Otorhinolaryngol. (2019) 123:33–7. 10.1016/j.ijporl.2019.04.037 31059930

[4] Honeyman JN Theilen TM Knowles MA McGlynn MM Hameed M Meyers P Desmoid fibromatosis in children and adolescents: a conservative approach to management. J Pediatr Surg. (2013) 48(1):62–6. 10.1016/j.jpedsurg.2012.10.017 23331794

[5] Pounds N Skapek SX. Desmoid-type fibromatosis in children: a step forward in the cooperative group setting. American Society of Clinical Oncology Educational Book. American Society of Clinical Oncology. Annual Meeting. (2012):593–7. 10.14694/EdBook_AM.2012.32.32 24451802

[6] Bhat V Raju P Rao S Ramaiah S. Infantile fibromatosis: a rare cause of anterior mediastinal mass in a child. J Clin Imaging Sci. (2015) 5:34. 10.4103/2156-7514.159452 26180657 PMC4490575

[7] Zhang Z Shi J Yang T Liu T Zhang K. Management of aggressive fibromatosis. Oncol Lett. (2021) 21(1):43. 10.3892/ol.2012.991 33262835 PMC7693298

[8] Makarawo TP Reynolds RA Cullen ML. Polylactide bioabsorbable struts for chest wall reconstruction in a pediatric patient. Ann Thorac Surg. (2015) 99(2):689–91. 10.1016/j.athoracsur.2014.03.052 25639409

[9] Risoud M Mortuaire G Leroy X Leblond P Fayoux P. Desmoid tumours of the head and neck in children: review of management. Eur Ann Otorhinolaryngol Head Neck Dis. (2017) 134(3):155–60. 10.1016/j.anorl.2016.11.007 27988199

[10] Greene AC Van Tine BA. Are the pieces starting to Come together for management of desmoid tumors? Clin Cancer Res. (2022) 28(18):3911–3. 10.1158/1078-0432.CCR-22-0620 35819317

[11] Desmoid Tumor Working Group. The management of desmoid tumours: a joint global consensus-based guideline approach for adult and paediatric patients. Eur J Cancer (Oxford, England: 1990). (2020) 127:96–107. 10.1016/j.ejca.2019.11.013 32004793

[12] Kasper B Raut CP Gronchi A. Desmoid tumors: to treat or not to treat, that is the question. Cancer. (2020) 126(24):5213–21. 10.1002/cncr.33233 33022074

[13] Riley DS Barber MS Kienle GS Aronson JK von Schoen-Angerer T Tugwell P CARE Guidelines for case reports: explanation and elaboration document. J Clin Epi. (2017) 89:218–35. 10.1016/jclinepi.2017.04.026 28529185

